# An insula-frontostriatal network mediates flexible cognitive control by adaptively predicting changing control demands

**DOI:** 10.1038/ncomms9165

**Published:** 2015-09-22

**Authors:** Jiefeng Jiang, Jeffrey Beck, Katherine Heller, Tobias Egner

**Affiliations:** 1 Center for Cognitive Neuroscience, Duke University, PO Box 90999, Durham, North Carolina 27708, USA; 2 Department of Psychology & Neuroscience, Duke University, PO Box 90086, Durham, North Carolina 27708, USA; 3 Department of Neurobiology, Duke University Medical School, Durham, North Carolina 27710, USA; 4 Department of Statistical Science, Duke University, PO Box 90251, Durham, North Carolina 27708, USA

## Abstract

The anterior cingulate and lateral prefrontal cortices have been implicated in implementing context-appropriate attentional control, but the learning mechanisms underlying our ability to flexibly adapt the control settings to changing environments remain poorly understood. Here we show that human adjustments to varying control demands are captured by a reinforcement learner with a flexible, volatility-driven learning rate. Using model-based functional magnetic resonance imaging, we demonstrate that volatility of control demand is estimated by the anterior insula, which in turn optimizes the prediction of forthcoming demand in the caudate nucleus. The caudate's prediction of control demand subsequently guides the implementation of proactive and reactive attentional control in dorsal anterior cingulate and dorsolateral prefrontal cortices. These data enhance our understanding of the neuro-computational mechanisms of adaptive behaviour by connecting the classic cingulate-prefrontal cognitive control network to a subcortical control-learning mechanism that infers future demands by flexibly integrating remote and recent past experiences.

## Abstract (web summary)

The ability to continually adjust behavioural strategies is a hallmark of human cognition, yet the underlying mechanisms remain poorly understood. Here the authors show that an insula-frontostriatal network mediates such flexible cognitive control by adaptively predicting changing control demands.

## Graphical abstract

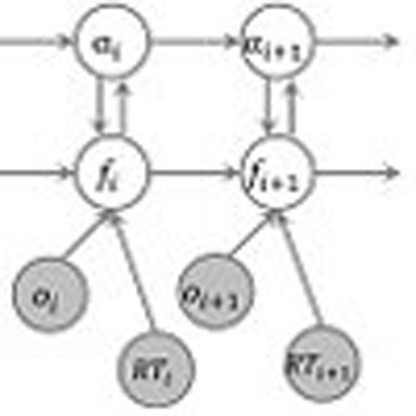

‘Cognitive control' describes the strategic guidance of behaviour in accordance with internal goals^1^. A key feature of cognitive control is flexibility, that is, the continual adjustment of processing strategies in response to varying demands. For instance, we are able to mobilize and adaptively shift attention towards a particular task when we encounter difficulties, such as worsening weather conditions during a road trip. Accordingly, laboratory studies have documented that human participants dynamically upregulate their attentional focus on task-relevant stimulus features in response to ‘conflict' induced by incongruent task-irrelevant stimulus features^2^,^3^,^4^. This type of online adaptation to varying control demand can be observed both as a function of short-term^5^,^6^,^7^ and long-term^8^,^9^ trial history, suggesting that the brain integrates information at multiple temporal scales to adjust conflict–control processes in line with anticipated future demands. However, the neuro-computational mechanisms supporting this integration remain poorly understood.

The influential conflict-monitoring model^2^ shows that short- and long-term adaptation to control demand can be simulated by a standard reinforcement learning mechanism that adjusts control (here, attentional focus on task-relevant stimulus information) to minimize the mismatch, or prediction error, between the level of control exertion and control demand. To simulate short-term adaptation, the model uses a fixed, high learning rate (LR), such that control adjustments are driven most strongly by prediction error from the most recent trials. To simulate long-term adaptation, the model employs a fixed, low LR, where control adjustments are driven by an extended trial history. This results in two key shortcomings, however: first, the use of fixed LRs means that the model, unlike people, lacks a mechanism to shift learning strategies when the nature of control demand changes over time. Second, while the model can successfully simulate short- and long-term adaptation effects in isolation, it is incapable of simulating them simultaneously, even though these effects do in fact co-occur in empirical data^10^,^11^. A more realistic account of human conflict–control would plausibly require the ability to flexibly self-adjust LRs in line with changing environmental contingencies.

To build such a model, we exploited recent advances in the modelling of Bayesian decision making under uncertainty, where it has been shown that LR adjustments can be derived from an estimation of the volatility (that is, rate of change) of the environment^12^,^13^,^14^. This is because, in a stable environment, LRs should be low to dampen learning, so that predictions are generated by integrating evidence over long periods of time. Conversely, in a volatile environment, LRs should be high so that predictions are generated only from recent observations and not from older, outdated evidence. On the basis of this perspective, we develop a hierarchical Bayesian extension of the standard reinforcement learning algorithm that includes a flexible control component to infer subjects' internal states related to flexible cognitive control using observed congruency and behaviour (Fig. 1a, lower panel, also see Methods). The top level in the hierarchy represents a volatility-driven flexible LR that quantifies the model's (or a subject's) belief concerning the rate of change of control demand (here, trial congruency) in the environment, which is learned on the basis of behaviour and the unsigned prediction error of congruency (control prediction error), quantified as the interaction between predicted conflict level and observed congruency (Supplementary Methods). This LR informs the middle layer of the model, which encodes the level of predicted conflict (or control demand), representing the belief of encountering an incongruent trial. Crucially, belief that the agent is in a higher volatility environment results in a larger LR during conflict prediction. Given this relationship, we will henceforth use the terms ‘volatility' and ‘flexible LR' interchangeably. However, this should not be interpreted as indicating that the objective task volatility is the same as a subject's LR. In fact, it has been shown that LR can be modulated by multiple factors, such as change point probability, and uncertainty about the environment and reward, each of which has distinct neural substrates^14^. Rather, ‘volatility' in the present model refers to the subjective belief that quantifies the factors that determine the learning rate.

The bottom level of the model represents the observable variables of congruency and reaction time (RT), which model the subject's experience of conflict and are, therefore, used to update beliefs about volatility and predicted conflict level at each trial. Note that within this prediction-updating scheme, we can distinguish two potentially distinct forms of control, a ‘proactive' component that corresponds to strategic processing adjustments in line with the model's prediction of control demand, and a ‘reactive' one that would be required to resolve any residual conflict if a mismatch (that is, control prediction error) occurred between predicted conflict and observed congruency^15^,^16^.

In prior work we have demonstrated that a Bayesian model with a flexible learning strategy can successfully track changing control environments and reproduce the simultaneous behavioural short-term and long-term adaptation effects observed in human performance^10^, thus supporting the proposal that the human cognitive apparatus estimates the volatility of control demand to optimize conflict prediction and cognitive control. We here combined a volatility-modulated conflict task with functional magnetic resonance imaging (fMRI) and model-based analyses to reveal the neural architecture underpinning these computations. We found that volatility of control demand and the prediction of forthcoming demand are estimated by the anterior insula and caudate nucleus, respectively. The prediction of control demand then guides the implementation of proactive and reactive attentional control in dorsal anterior cingulate cortex (ACC) and dorsolateral prefrontal cortices. These data enhance our understanding of the neuro-computational mechanisms of adaptive behaviour by connecting the classic cingulate-prefrontal cognitive control network^17^ to a subcortical control-learning mechanism that infers future demands by flexibly integrating remote and recent past experiences.

## Results

### Task overview

fMRI data were acquired while subjects (*N*=21) performed a face-word Stroop conflict task^6^,^10^,^18^, in which they responded to the gender of face images via button-presses and tried to disregard overlaid gender word labels (Fig. 2a), which could be either congruent (for example, ‘male' superimposed on a male face) or incongruent (for example, ‘female' superimposed on a male face). To investigate the flexible adjustment of cognitive control, we furthermore varied the relative dependence on short-term versus long-term trial history required for achieving optimal prediction of control demand. In the *volatile* control demand condition, the trial sequence sampled alternately from distributions with a 20 or 80% proportion of incongruent trials, switching every 20 trials over a run of 80 trials. Conversely, in the stable control demand condition, the underlying proportion of incongruent trials remained unchanged (either 20 or 80%) for a run of 80 trials. In the volatile condition, more frequent change of the proportion of incongruent trials (that is, control demand) should encourage a higher LR in the prediction of conflict, as compared with the stable condition. Note that the overall incidence of congruent and incongruent trials was equal (0.5) across stable and volatile runs (see Methods).

### Behaviour

Descriptive statistics of behaviour are presented in Supplementary Table 1. Subjects performed the volatility-modulated conflict–control task with high accuracy (mean accuracy=94.2%). A three-way analysis of variance (ANOVA; volatility × proportion of incongruent trials × congruency) revealed a main effect of congruency (*F*_1,20_=9.15, *P*<0.005), due to higher accuracy in congruent (94.9%±1.2) than incongruent (93.1%±1.0) trials, as well as a main effect of proportion of incongruent trials (*F*_1,20_=4.77, *P*<0.05) due to higher accuracy in blocks of 80% incongruent trials (94.8%±1.0) than blocks of 20% incongruent trials (93.2%±1.2). In RT data, a three-way ANOVA detected a significant main effect of congruency (*F*_1,20_=20.4, *P*<0.0001), driven by a slower RT in incongruent trials (458 ms) than congruent trials (418 ms).

### Model validation

We used the flexible control model to simulate behavioural data on a trial-by-trial basis (see Methods). For each subject, observed congruency and response speed (*RS*, defined as 1/*RT*) were used to infer trial-by-trial estimates of the volatility-driven flexible LR and predicted conflict level. As expected, the model estimate of LR was indeed higher for volatile than for stable runs (Fig. 2b, paired *t*-test, *t*_20_=21.22, *P*<0.0001). In both volatile and stable blocks, the LR increased soon after the underlying proportion congruency changed, and gradually decreased as the model learnt the proportion congruency of the new environment (Fig. 2c). These results strongly suggest that the model estimates of flexible LR are sensitive to the structure of the task. Moreover, the model estimates of the flexible LR resemble a previous simulation study of the proportion congruency effect^2^. Finally, the estimates of predicted conflict level tracked the time course of the underlying proportion congruency very closely (Fig. 2d). These results indicate that the model beliefs successfully tracked the experimental manipulations. We next employed three model variables (trial-wise estimates of volatility and predicted conflict level, and the observed congruency) and their interactions (that is, a total of seven variables, resembling a three-way ANOVA) to account for the variance in RS (see Methods). This analysis revealed that, unsurprisingly, the congruency variable was a strong predictor of RS, with slower responses associated with incongruent trials (*t*_20_=4.17, *P*<0.001, Fig. 2e). More importantly, and reflecting the basic tenet of our model, the larger the model's trial-wise control prediction error was, the slower were the participants' responses, as reflected in a negative modulation of control prediction error on RS (*t*_20_=−2.79, *P*=0.01, Fig. 2f). This result is unlikely to be biased by the use of RS to update the model's beliefs, because we did not constrain the manner in which RS could correlate with the model variables, like predicted conflict level, to best account for behaviour. In sum, the flexible control model closely captured behaviour in a conflict task with time-varying control demands.

### Model comparison

The fact that a conflict–control model with a volatility-driven flexible LR can explain participants' behaviour in an environment of changing control demands does not imply the necessity of a flexible LR in explaining behaviour. We therefore conducted a model comparison that pitted the flexible control model against simpler reinforcement learning models with one learning rate (for the whole task) or two learning rates (one for stable runs and one for volatile runs). Model performance was quantified using the Bayesian information criterion (BIC; see Methods). The best LRs for fixed-LR models were selected based on an exhaustive search (from 0.01 to 0.5 with a step size of 0.001) that minimized the BIC. For each subject, the models were fit to the individual trial sequence of congruency and RS, and the simulated RS values derived from each model fit were then used to calculate the BIC, on which the model selection was based. A group level model comparison^19^ revealed an exceedance probability (that is, the belief that one model explains data better than other models in the comparison) of 0.99 for the flexible control model (Supplementary Table 2). To further test whether the flexible control model is also superior at predicting forthcoming congruency, we repeated the model comparison analysis using BIC calculated from candidate models' prediction of conflict in relation to observed congruency. The same flexible control model as above was used, whereas the fixed-LR models were re-selected using exhaustive search to ensure that the fixed-LR models that best accounted for congruency were employed in this analysis. A group level model comparison^19^ revealed an exceedance probability of 0.91 for the flexible control model (Supplementary Table 3). Additionally, note that the best-fitting LRs in the fixed-LR models are determined in a *post hoc* manner, which is not a luxury the brain has. By contrast, the flexible control model does not benefit from *post hoc* fitting, as it relies solely on the LR as it emerges from the model's interaction with the trial sequence experienced so far. On the basis of these results, we accepted the flexible control model as the best account for participants' behaviour and task manipulation in this volatility-modulated cognitive control task.

### Assessing neural computations of flexible control

Next, we sought to determine the neural substrates of the flexible control model's components, namely, the estimation of control demand volatility (flexible LR), the prediction of conflict (control demand), and the application of proactive control as a function of that prediction; moreover, based on the residual of that prediction, we can infer demands on reactive control mechanisms. The vector of ‘congruency' in and of itself was not of interest, as it simply coded for observed trial congruency. Brain regions that were more activated by incongruent than congruent stimuli replicated standard findings in the literature^20^ (Supplementary Fig. 1).

**Figure 1:**
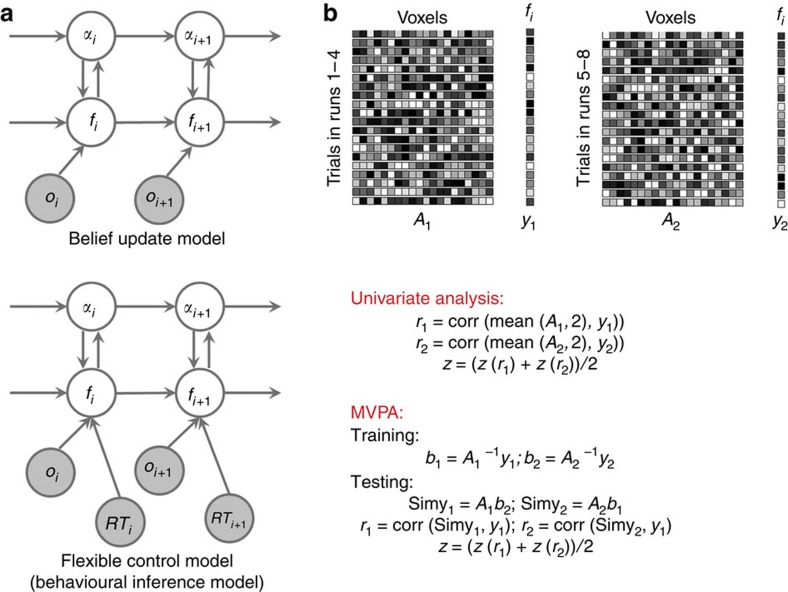
Model structure and schematic illustration of model-based analyses (*N*=21). (**a**) The graphical representation of the belief update model (upper panel) and the flexible control model (lower panel). The two models use identical structure and inference algorithms except that the flexible control model also uses reaction time (RT) to update the belief of latent variables. Note that only the flexible control model was used in all analyses to account for individual difference of behaviour. The flexible control model uses four variables, flexible LR/volatility (*α*), predicted conflict (*f*), congruency (*o*, shown in grey indicating this variable is observable) and RT for each trial. The directed edges indicate the information flow. At a given trial, horizontal and top–down edges represent the estimation of flexible LR/volatility and predicted conflict level prior to stimulus presentation. Subsequently, the observed congruency and RT are used to update belief of latent variables in anticipation of the next trial (bottom-up edges). (**b**) A schematic illustration of the univariate and multivariate encoding analysis using the example model variable of predicted conflict level (see Methods for details). Importantly, prior to multivariate analysis, the searchlight mean activation vector was also regressed from each voxel's activation vector to ensure the orthogonality between the two analyses.

For delineating neural substrates of model components, we assessed whether local trial-by-trial fluctuations in neural activity (univariate or multivariate activation vectors) could explain a significant portion of trial-by-trial variance in the inferred states of model variables. To this end, we performed parallel univariate and multivariate searchlight analyses (radius=2 voxels)^21^,^22^, which accounted for the possibility that the variables in question could be encoded either via a homogeneous (univariate) scheme (Fig. 1b), where information is encoded by local voxel populations with similar response properties (for example, a group of voxels whose fMRI signal amplitudes all scale positively with predicted conflict), or via a heterogeneous, distributed scheme, where information is encoded in multivariate activation patterns over local voxel populations^21^ (Fig. 1b, also see Methods). Within small clusters of voxels (that is, the searchlight), homogeneous activation patterns will be better detected by the univariate analysis, whose averaging process (see below) boosts the signal-to-noise ratio of the homogeneous activation patterns. By contrast, local heterogeneous activation patterns, where, for example, each voxel encodes a different aspect of the variable of interest, or only a few voxels accurately encode the variable of interest while other voxels are not sensitive to that variable, will be more easily identified by the multivariate analysis. To ensure the unique attribution of fMRI signal to a given variable (or interaction among variables), all other variable and interaction vectors were used as nuisance variables in both analyses. A summary of all findings from these analyses is available in Supplementary Table 4.

### Anterior insula estimates volatility of control demand

Beginning with the top-level model node, we first conducted a whole-brain search for regions that encoded the flexible LR (that is, volatility), by fitting searchlight activation vectors to the (residual) flexible LR variable vector (*α*). We found that the bilateral anterior insula (AI) and adjacent inferior frontal gyri (IFG), the amygdala, putamen and right parahippocampal gyrus and precuneus (Fig. 3a; *P*<0.05, corrected) all tracked this model variable vector via a homogenous (univariate) coding scheme, reflected in higher signal amplitudes for higher LR estimates. These areas therefore represent candidate regions for encoding control demand volatility. We next performed an additional, independent analysis to cross-validate this hypothesis.

Recall that after congruency is observed, the model's LR needs to be updated to guide the adjustment of predicted conflict level (Fig. 3b), based on the control prediction error (predicted conflict level × congruency interaction). Given that the control prediction error is required for updating the LR, we reasoned that brain regions that compute the LR of control demand would likely also harbour a representation of control prediction error. We therefore conducted another whole-brain search for regions whose activation vectors reflected this interaction, which revealed a cluster of searchlights in the left AI/IFG (Fig. 3c; *P*<0.05, corrected). This cluster overlapped closely with the AI/IFG region encoding the volatility/LR model vector (Fig. 3a), within which 28 out of 77 searchlights also showed encoding (*P*<0.05) of control prediction error (Fig. 3d). Accordingly, across the searchlights in the left AI/IFG encoding the belief of control demand volatility (Fig. 3a), the mean effect of control prediction error was significant (*t*_20_=−1.97, *P*<0.05).

To further characterize the role of this region in representing the LR of control demand predictions, we plotted model LR against neural activation estimates (Fig. 3e), where a strong linear relationship is observed. One possible rival interpretation of these data could be that the AI/IFG signals reflect uncertainty rather than volatility of predicted conflict, as uncertainty and volatility tend to be highly correlated^12^. To rule out this possibility, we repeated our model-based analysis while replacing flexible LR with ‘estimation uncertainty' (calculated as the s.d. of the distribution of predicted conflict level). We did not observe an effect of estimation uncertainty of conflict in the AI/IFG. This selective encoding of the flexible LR in this task can be attributed to the fact that within each subject the trial-wise model estimates of these two variables in stable and volatile blocks only shared a relatively small proportion of variance (Supplementary Tables 5 and 6; Fig. 3f). This distinction is also consistent with previous findings showing dissociable neural signatures of volatility and estimation uncertainty^12^. However, future studies are required to determine the exact relationship between LR and estimation uncertainty in a broader context. Taken together, we obtained strong evidence that the (left) AI and IFG (Brodmann areas 13 and 47) encode and update a volatility-driven flexible LR for predicting control demand in a non-stationary environment.

### Caudate nucleus predicts control demand based on volatility

In the flexible control model, the volatility-driven flexible LR informs the prediction of control demand (conflict). We therefore next sought to identify the neural substrates of conflict prediction, by conducting a whole-brain search for brain regions whose activation vectors could account for significant variance in the (residual) conflict prediction variable vector (*f*). We found that the body of the (right) dorsal caudate nucleus tracked the model's predicted conflict levels (Fig. 4a; *P*<0.05, corrected) via a heterogeneous (multivariate) coding scheme (Fig. 1b). Additionally, predicted conflict levels were also encoded in a homogeneous (univariate) fashion in the left inferior parietal lobule, left superior frontal gyrus and right paracentral lobule, where larger predicted conflict levels were associated with higher activity. We next performed an additional, independent analysis to cross-validate these putative neural substrates of volatility-driven updating of predicted conflict.

Before stimulus presentation in a given trial, the flexible control model updates predicted conflict level by integrating the most recently observed congruency, modulated by the model's LR (Fig. 4b), an effect we should also expect to observe in a conflict-predicting brain region. To test whether any of the above reported regions displayed this type of modulation, we extracted a ‘neural LR', which was derived from these regions' activation vectors without any assumption of the flexible control model, and we then examined the linear relation between neural LR and the model LR for each subject (Supplementary Methods). A significant positive scaling between neural LR and model LR was observed in the caudate (Fig. 4c, *t*_20_=4.70, *P*=0.0001), supporting the idea that the volatility-driven flexible LR modulates the updating of conflict predictions in this region. By contrast, the equivalent analyses in other regions did not produce significant results. In sum, the above results provide strong evidence for the caudate to be involved in the computation of (volatility-modulated) predicted conflict levels. These predicted conflict levels provide guidance for the implementation of flexible cognitive control, whose neural substrates were investigated in the following analysis.

### Proactive and reactive cognitive control in prefrontal cortex

The behavioural results documented that larger control prediction errors (that is, the discrepancy between predicted and actual conflict level) are associated with slower RS, which is in line with the basic model assumption whereby the implementation of cognitive control is guided by predicted conflict level and subsequent control prediction error. In a final set of imaging analyses, we aimed to link back directly to this behavioural performance pattern by examining how the volatility-driven prediction of control demand was translated into the recruitment of brain regions that mediate the application of proactive and, in the case of erroneous predictions, reactive cognitive control, within a ‘dual mechanisms of control' framework^16^ (Fig. 5a). These analyses thus employ the individual behavioural correlation between control prediction error and RS as the to-be-explained target variable, and ask in which brain regions the neural encoding of the model variables of control demand prediction and control prediction error may explain variance in this target variable. To this end, we employed an individual difference analysis that linked variance in encoding strength of model variables to variance in the target variable. Specifically, since proactive control is applied based on predicted conflict, the level of proactive control should be positively correlated with the target variable, because stronger proactive control should improve performance (that is, produce faster RS) when the observed control demands closely match predictions (that is, lower control prediction error), resulting in a greater effect of the target variable. Thus, to identify the neural substrates of proactive control, we tested the correlation between the target variable (obtained from the behavioural analysis above) and encoding strength of predicted conflict level (obtained from the imaging analysis) across subjects, at each searchlight. We detected this signature of proactive control in the caudal ACC (Fig. 5c, red overlay), and left dorsolateral prefrontal cortex (dlPFC, Fig. 5b, red overlay). Here, subjects showing a stronger neural encoding of predicted conflict level displayed a greater effect of control prediction error in RS, indicating a direct role for these regions in translating predicted control demand into adaptive processing strategies.

According to the dual-mechanism model, after the imperative stimulus is observed, reactive control must be recruited in proportion to the necessity of resolving any residual conflict, that is, the prediction error arising from a mismatch between predicted and actual control demand^16^. Stronger reactive control should lead to more efficient resolution of residual conflict, which in turn should translate into a smaller effect of control prediction error in RS. In other words, since reactive control is assumed to counteract the consequences of a mismatch between predicted conflict (proactive control) and observed congruency, reactive control strength should be negatively correlated with the target variable. Thus, to identify the neural substrates of reactive control, we tested the correlation between the target variable and encoding strength of control prediction error (obtained from the imaging analysis) across subjects, at each searchlight. We detected the hypothesized signature of reactive control in the rostral ACC (Fig. 5c, green overlay). Here, subjects showing a stronger neural encoding of control prediction error displayed a weaker effect of control prediction error in behaviour, indicating a close involvement of this region in the process of reactively resolving unanticipated processing conflict. In sum, these two analyses revealed brain regions that appear to be involved in either proactive or reactive cognitive control, which jointly mediate conflict resolution in the context of predicted control demand, as reflected in the behavioural performance pattern where larger control prediction error is associated with slower responses.

## Discussion

Previous work has shown that conflict prediction based on a fixed LR can capture human adaptive behaviour in stationary environments, and has implicated the dACC and dlPFC in mediating conflict–control^2^,^17^. Here we showed that the flexible adaptation of cognitive control to a dynamically changing environment can be achieved by self-adjusting, volatility-weighted reinforcement learning of control demand, and that this dynamic form of cognitive control depends on learning processes that have neural correlates in the AI/IFG and dorsal striatum. Specifically, model-based fMRI analyses documented that the activity in the AI adjacent IFG tracks a flexible, volatility-driven LR; this flexible LR can be employed to optimize the prediction of forthcoming control demand (conflict), a prediction we found to be encoded in activation patterns of the caudate. This prediction in turn should guide the implementation of proactive control to match the anticipated conflict, and we observed correlational evidence to suggest that this process is carried out by the dACC and the dlPFC; the residual of that conflict (control prediction error) appears to be resolved by a more rostral portion of the ACC.

Both the volatility (flexible LR) of control demand and control prediction error were found to be encoded by the AI/IFG, suggesting that this region dynamically maintains an estimate of the degree to which current control demands are stable or fast-changing. The association of this area with an explicit computation of control demand volatility promotes a new, integrative interpretation of results from a diverse collection of previous studies. In broad agreement with the present findings, the AI/IFG have long been argued to form part of a fronto-parietal ‘multiple demand' cognitive control network^23^,^24^, with some authors postulating a more specific role of a cingulo-opercular sub-network in sustaining ongoing task control^25^,^26^, based on these regions' tendency to be (tonically) more activated in conditions of higher relative to lower task demands^23^,^24^,^25^,^26^.

Moreover, neuroeconomics studies have tied the AI to representing the uncertainty of reward location^14^, reward magnitude^27^ and risk prediction error^28^. Of particular relevance, it has been shown that the AI displays higher activation during decision-making involving ambiguity (an unknown probabilistic distribution of reward) than that involving risk (a known probabilistic distribution of reward)^29^. These results closely align with our findings, because volatility quantifies the ambiguity of the belief of the probabilistic distribution of conflict. Thus, the present model and findings may provide a formal account explaining why this region has previously been implicated in the computation of risk prediction error.

Note, however, that one previous study has attributed the computation of volatility of reward likelihood to the dACC^12^. Different findings between the two studies should not be surprising though, because they focused on distinct cognitive processes. Our task required the learning of abstract control demands, whereas that study involved the learning of specific stimulus-action-reward associations. In addition, the present fMRI analysis assessed the activity in relation to the processing of the imperative (to-be-presented) stimulus, whereas that analysis^12^ focused on the post-outcome (explicit reward feedback) period. The two studies' results are therefore not directly comparable.

Moving from volatility to anticipating control demand, we found that the flexible LR informed a representation of predicted conflict in the dorsal caudate body. Specifically, the caudate employed volatility estimates to fine-tune the integration weights of temporally more recent versus more remote control prediction errors in the service of predicting forthcoming control demand. This region is anatomically connected to the prefrontal cortex^30^ and thought to form part of a cognitive or ‘associative' frontostriatal loop^31^ mediating the selection or gating of information to be represented in task-sets held by the prefrontal cortex^32^,^33^,^34^. Aligned to this perspective, the present data foster the proposal that the caudate's contribution to cognitive control may be to optimize frontostriatal gating processes by predicting forthcoming control demand, which could inform both the selection and vigour of rule representations in frontal cortices. Of note, we found predicted conflict level to be encoded via a distributed (multivariate) coding scheme, which suggests that the expected control demand is represented across distinct local neuronal populations, with each group's activation tuned to respond to a specific level of predicted conflict level^35^. In concordance with this finding, the parallel circuit organization of the caudate^36^,^37^ can be argued to provide a feasible structural foundation for representing a statistical variable.

Finally, leaning on the dual-mechanisms-of-control framework^16^, the present study also allowed us to formally tease apart, within the same trial, neural substrates of reactive control from those of proactive control. In the prior literature, two possible dual-mechanism architectures have been discussed; one assumes proactive and reactive control to be implemented via different temporal activation dynamics within the same frontal regions^38^, and the other postulates the existence of distinct brain regions supporting the two types of control^39^. Our results provide novel evidence for the latter proposal, as we found distinct brain regions showing neural signatures supporting either anticipatory or reactive control. On the one hand, we found that the caudal dACC and dlPFC (Fig. 5b,c) displayed neural profiles commensurate with a role in supporting anticipatory control guided by predicted conflict levels. This finding broadly recapitulates a large prior literature implicating these regions in mediating trial-by-trial conflict-driven adjustments in control^5^,^6^,^7^,^40^, though the present data enrich this perspective by documenting that this frontal conflict–control network is tuned to changing control environments via control-learning signals computed in the insula and caudate. On the other hand, we detected a focus in the most anterior portion of the rostral cingulate zone^41^ selectively involved in reactive control processes (Fig. 5c), as indicated by activity that scaled with the successful resolution of residual (unpredicted) conflict. While a general involvement of this rostral ACC territory in conflict–control processes has been suggested by some previous studies^42^,^43^, a role specific to the resolution of residual control prediction error has not been articulated and merits more in-depth exploration in future work. Note that the manner in which we identified this rACC region makes the assumption that a higher degree of reactive control should be associated with better resolution of residual conflict. This type of reactive control signature implies that the rACC plays a role in dampening the influence of unexpected incongruent distracters on response selection, resulting in faster responses. An alternative type of reactive control mechanism could respond to unexpected conflict by raising the response threshold, to delay response execution until conflict has been resolved. Here, higher reactive control would be associated with slower (but more accurate) responses. Interestingly, this type of reactive control signature has been attributed to interactions between a more dorsal ACC region and the subthalamic nucleus^44^. Side-by-side, these findings raise the intriguing possibility that different ACC territories may be involved in two different, complementary reactive control processes, which could be tested empirically in future studies.

To conclude, while flexibility in response to changing environments has long been thought of as a defining characteristic of cognitive control, the present study is, to the best of our knowledge, the first to document the neuro-computational mechanisms underlying the flexible adaptation of control to time-varying demands. Extending the basic conflict-prediction framework^2^, we show how changing control demand contingencies can be effectively learned to predict conflict and adjust cognitive control via an adaptive, volatility-driven LR. Delineating the neural substrates of flexible control, we found that the activity in the AI/IFG reflects a self-adjusting, volatility-modulated LR that informs the adaptive prediction of anticipated control demand, which appears to involve the caudate; the predicted control demand in turn proactively guides control to match anticipated needs, likely via the ACC and dlPFC regions; prediction failure requires reactive control, which appears to rely on the rostral ACC.

## Methods

### Subjects

Twenty-one healthy, right-handed volunteers (eight females, mean age=26 years) gave informed consent in accordance with institutional guidelines. All subjects were native or highly proficient English-speakers and had normal or corrected-to-normal vision. This study is approved by the Duke University Health System Institutional Review Board. The sample size was determined based on a previous analysis^45^.

### Apparatus and stimuli

Stimulus delivery and behavioural data collection were carried out using Presentation ( http://www.neurobs.com/). Stimuli consisted of a collection of 24 grey-scale photographs of male and female faces (12 each) of neutral expression that were overlaid with red gender word labels (‘man', ‘woman', ‘male' and ‘female'), which could be printed in lower or upper case lettering. Visual stimuli were presented on a back projection screen viewed via a mirror attached to the scanner headcoil. On each trial, one face-word compound stimulus (subtending ∼3° of horizontal and 4° of vertical visual angle) was presented against a grey background in the centre of the screen.

### Procedure and task design

Stimuli were presented for 250 ms, followed by a jittered inter-stimulus interval ranging from 4 to 6 s in uniformly distributed steps of 500 ms, during which a fixation cross remained on screen. Subjects performed a speeded button response that categorized the gender of the face stimulus with either index finger (for example, left-hand response to male faces, right-hand response to female faces, counterbalanced across subjects), while trying to ignore the task-irrelevant gender labels. Responses were collected using an MRI-compatible button box. To preempt low-level priming effects, face stimuli never repeated across adjacent trials, and the lettering alternated between lower- and upper-case across trials. A practice run was conducted before the subjects entered the MRI scanner to ensure they comprehended the task requirements.

The task consisted of eight runs of five blocks each. The first block contained 16 trials and had 50% congruent trials and served as a burn-in block to bring predictions to the same baseline at the beginning of each run. The remaining four blocks contained 20 trials each. To create experimental environments that differ in their dependence on long-term and short-term trial history, a run could be either volatile (the proportion incongruency altered between 20 and 80% every block) or stable (the proportion incongruency remained either 20 or 80% for all four post-burn-in blocks), though overall, the proportion of incongruent trials was equal between stable and volatile runs (50% congruent and 50% incongruent trials, averaged over runs). The order of volatile and stable runs was counter-balanced across subjects. This manipulation resulted in a 2 (volatile/stable) × 2 (proportion incongruency) × 2 (congruency) factorial design.

### Behavioural data analyses

Performance accuracy and RT were analysed using a repeated-measure three-way ANOVA based on the factorial design. Error trials, post-error trials, outlier trials (RT values that deviated >2.5 s.d. from an individual subject's grand mean), and post-outlier trials were excluded from the RT and fMRI analyses, as these trials likely involve cognitive and/or affective processes additional to the computations of interest in this study. Specifically, error trials reflect either unsuccessful conflict resolution processes or lapses in task-set, and errors are accompanied by negative affective responses^46^, all of which represent possible confounding factors in our quest to model successful cognitive control over conflict. Similarly, trials following an error are known to display ‘post-error slowing', possibly due to a cautionary shift in response thresholds^47^,^48^, which represents a process that is not targeted in the current version of the flexible control model. Outlier and post-outlier trials were excluded from the analysis because they reflect rare extreme lapses in performance (and subsequent recovery processes) that are not representative of the typical cognitive control regulation we attempted to characterize. In addition, a model-based, trial-based analysis was performed. Specifically, individual sequences of trial congruency (concatenated across runs, with all trials included) and RS (1/RT) were processed by the flexible control model (see below) to generate trial-based estimates of LR and predicted conflict level, defined as the mean of the variables. Variable estimates and congruency for excluded trials were then discarded. The remaining estimates were grouped into a chronological vector for each model variable. As shown in Supplementary Tables 5 and 6, even though the some variables/conditions were significantly correlated with each other, the model variables/conditions did not share much variance, likely due to the high degrees of freedom (∼600 trials per subject). These vectors were then normalized and multiplied to form seven variable vectors (flexible LR/volatility, predicted conflict level, congruency, and their two-way and three-way interactions). Subsequently, these vectors were grouped, along with a constant vector, to form a general linear model. To test the effect of each of the seven variable vectors while ensuring that effects were not confounded by shared variance with any of the other variables, the ‘test variable' vector was first regressed against the other variable vectors. The residual resulting from this regression was then fit as a predictor to the RS vector, along with the other six variable vectors in the general linear model as nuisance effects. The resulting fitting coefficient for the test variable vector was then tested against 0 using a one-sample *t*-test across subjects.

### Flexible control model

Structurally, the flexible control model was based on the algorithm in Behrens *et al*.^12^. In the present study we re-describe this model as an extension of the conventional reinforcement algorithm to ensure biological plausibility and facilitate comparison with standard fixed-LR models. The model has four variables (Fig. 1a, lower panel), namely (1) the (volatility-driven) flexible LR (*α*) that quantifies the model's (or a subject's) belief concerning the rate of change of control demand (here, trial congruency) in the environment; (2) predicted conflict (*f*) that represents the believed probability of encountering an incongruent trial; (3) congruency (*o*) and (4) RT. Congruency and RT are observed variables that are included to infer the hidden model belief states of the LR and predicted conflict level. The inclusion of RT for inferring internal model states enables the model to account for individual difference in performing this task. For example, two subjects experiencing the same sequence of congruency but with different RT will produce different estimates of internal belief states. Each column in Fig. 1a represents the state of the variables in a given trial. In brief, the model makes predictions of congruency for the forthcoming trial, and the observed congruency and RT are used to infer model beliefs of flexible LR and conflict predictions, which are then used to make predictions for the next trial. How the distribution of each variable is determined by other variable(s) and/or parameter(s) is explained in detail below. The legitimacy of the model components were validated using model comparison (Supplementary Methods).

**Figure 2:**
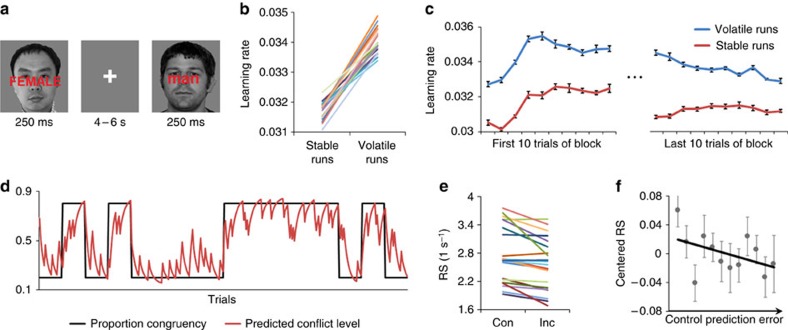
Experimental task and simulation and behavioural results (*N*=21). (**a**) Example stimuli and timing of presentation. This example depicts an incongruent trial, followed by a congruent trial. (**b**) Individual mean model LRs, plotted as a function of run type. Each line represents a subject. (**c**) The time course of group mean LR and s.e.m. in the first and last 10 trials of volatile (in blue) and stable (in red) blocks. Note that in this graph, which averages over all blocks, the difference in LR at the beginning of the blocks was driven by volatile blocks 2–4 in the volatile runs, as in these blocks the LR had already been raised by preceding volatile blocks. (**d**) Time courses of the underlying proportion congruency (in black) and the corresponding predicted conflict level (in red) of an example run. (**e**) Individual RS, plotted as a function of congruency. Each line represents one subject. Note that higher RT equals lower RS. (Con=congruent trials; Inc=incongruent trials). (**f**) Group mean RS and s.e.m., centred across trials for each subject, plotted as a function of unsigned prediction error of congruency (control prediction error).

**Figure 3:**
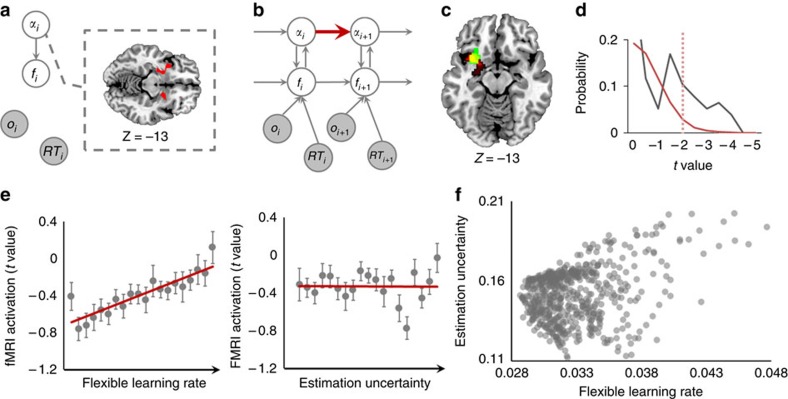
Estimation and updating of the volatility-driven flexible LR of control demand (*N*=21). (**a**) Searchlights in the AI and adjacent IFG track the model estimates of volatility/LR (in red, *P*<0.05 corrected, one sample *t*-test). (**b**) The flexible control model, highlighting in red the information processing mechanisms related to the updating of LR. (**c**) Visualization of searchlights encoding LR (red, *P*<0.05 corrected) and searchlights showing an interaction between predicted conflict level and congruency, or control prediction error (green, *P*<0.05 corrected, one sample *t*-test); overlap between these independently defined effects is shown in yellow. (**d**) Probabilistic distribution of *t*-values measuring the group-level univariate effect of prediction error of congruency (in grey) and the underlying null *t*-distribution (in red). The *t*-values were calculated from searchlights in the LR-encoding cluster shown in (**a**). The red vertical dotted line denotes the threshold for statistical significance (*P*<0.05). (**e**) Group mean activation levels (±s.e.m.) in the left AI/IFG ROI showing significant encoding of the volatility-driven flexible LR. The trial-by-trial ROI-mean fMRI activation (in *t*-values, in accordance with the univariate and multivariate analyses) were binned for each individual into 20 quantiles, which are plotted as a function of 20 quantiles of the LR (left) and estimation uncertainty (right). (**f**) Trial-wise estimation uncertainty of a representative example subject, plotted as a function of its corresponding LR. Each trial is represented by a translucent grey disk. Although the correlation coefficient between these two model estimates is significant (*r*=0.32, *P*<0.0001), the *r*^2^ statistic indicates that only 10% of variance is shared between them (for group statistics, see Supplementary Tables 5 and 6).

**Figure 4:**
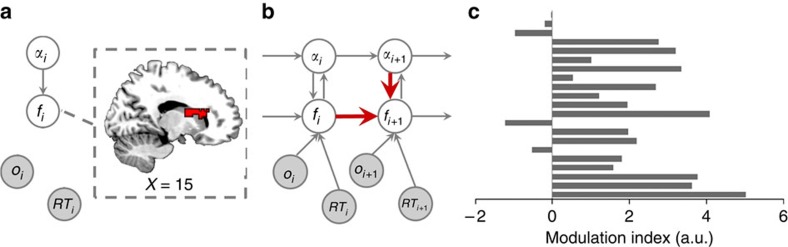
Modulation of volatility on predicted control demand (conflict level, *N*=21). (**a**) Searchlights in the caudate track the model's prediction of conflict level (in red, *P*<0.05 corrected, one sample *t*-test) (**b**) The flexible control model, highlighting in red the information processing mechanisms related to the modulation of volatility on predicted conflict level. (**c**) Individual modulation of volatility on caudate activity-derived LR. Each horizontal bar represents a participant.

**Figure 5:**
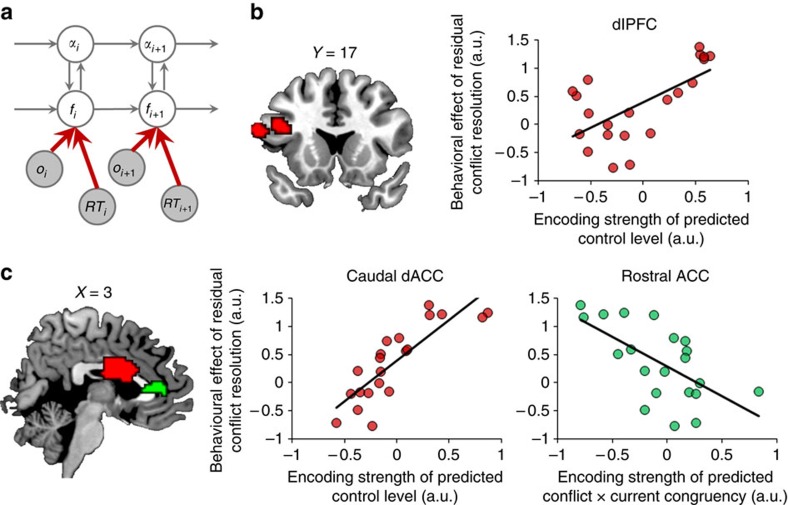
Titration of cognitive control by predicted conflict level (*N*=21). (**a**) The flexible control model, highlighting in red the information processing mechanisms related to the mediation of predicted conflict level on cognitive control. (**b**,**c**) Brain regions involved in proactive/reactive control in the dlPFC (**b**) and ACC (**c**), showing a significant correlation between predicted conflict level × congruency interaction in the RSs and encoding strength of predicted conflict level (in red)/prediction error of predicted conflict level (in green), respectively. For each cluster displayed, predicted conflict level × congruency interaction in the RS is plotted as a function of cluster mean of encoding strength across subjects.

The transition of the flexible LR is defined to most likely remain in its previous state; if it changes state, however, it is equally likely to jump to any other value (that is, following a uniform probability distribution):


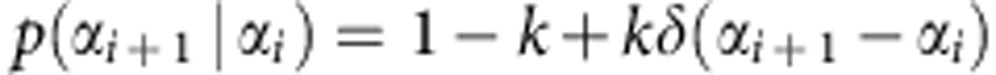


where 0<*α*_*i*_, *k*<1. This definition captures the fact that, in real-life environments (and in our task manipulation), at any given instance control demands are more likely to stay the same than to change, which in our task is captured by the fact that at any given trial transition, the proportion congruency is more likely to stay the same than to jump to another value. Compared with other change point detection algorithms, this transition function is more feasible because it does neither require *a priori* knowledge of the rate of change of proportion congruency^49^, which is not disclosed to the subjects in our experiment, nor a perfect memory of all previous trials^50^. Note that we did not hard-code the task structure (for example, the underlying proportion congruency changes every 20 or 80 trials) in the flexible control model. This is due to two reasons: first, unlike a normative model that has explicit knowledge about how LR should behave, we aimed to employ an agnostic model with a uniform prior to infer how one's belief of LR changes using observed congruency and behaviour. Second, the manipulation of volatility was not disclosed to the subjects before finishing the task. Subjects were therefore unlikely to have strong, specific expectations concerning forthcoming changes in task volatility (that is, change of the underlying proportion congruency). As a consequence, a uniform prior was chosen for the model's prediction of the LR at the forthcoming trial. However, the choice of a uniform prior does not imply that the flexible LR is insensitive to the underlying change of proportion congruency. As can be seen in Fig. 2c, the flexible LR increases soon after a change in proportion congruency (that is, at the beginning of blocks), indicating that the LR variable, using a uniform prior, adapted to the change in the proportion of congruency, with increases in LR in a more volatile environment, and decreases in a more stable environment.

Given the randomness of the sequence of congruency in our experiment, it is impossible to make a precise prediction of conflict (for example, predicting conflict level to be 0.8 with 100% certainty). Hence the prediction should be approximate, leading to a smooth distribution of predicted conflict level. For example, high likelihood of the predicted conflict level being 0.8 should also imply high likelihood of predicted conflict level at values close to 0.8. Hence the belief of the predicted conflict level being a particular value is propagated to nearby values. Because the (un)certainty of the prediction depends on volatility (for example, given that the prediction of the conflict level is 0.8, the distribution of true conflict level should surround 0.8 more tightly when the volatility is lower), we further used the volatility variable to control the propagation process in the following manner:


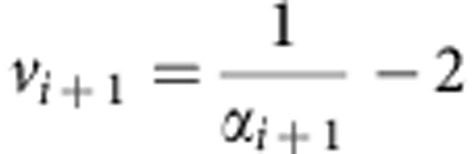



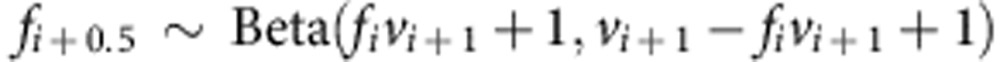


Here, the mode of the beta distribution is *f*_*i*_. The sum of the two parameters in the beta function is 
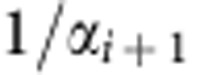
. Note that this sum modulates the width of the beta distribution (a higher sum results in a narrower beta distribution)^12^. Thus, a belief of lower volatility (that is, lower LR) would result in a narrower propagation distribution to simulate the distribution of true conflict level conditioned on the predicted conflict level. After propagation, the predicted conflict level is updated based on inverting the generative model under the assumption that the subject only updates his beliefs based upon the observation of conflict. This belief update has the form of a standard reinforcement learning rule with *α*_*i*+1_ playing the role of learning rate:


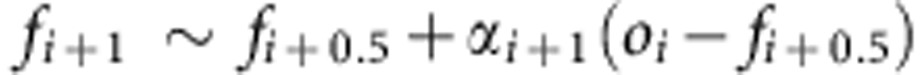


Note that we slightly abuse notation by letting *v*_*i*+1_ and *f*_*i*+1_ represent the mean of our belief distributions over these quantities in the behavioural and fMRI analysis. This belief update model describes how the subject makes his predictions based on observed conflict, and forms the core of our behavioural model. However, when attempting to infer a subject's belief we employed the RT. Therefore, when fitting the behavioural model our estimates of the subject's belief of *v*_*i*+1_ and *f*_*i*+1_ were updated in the following manner:


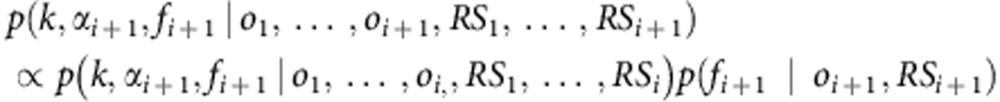


where


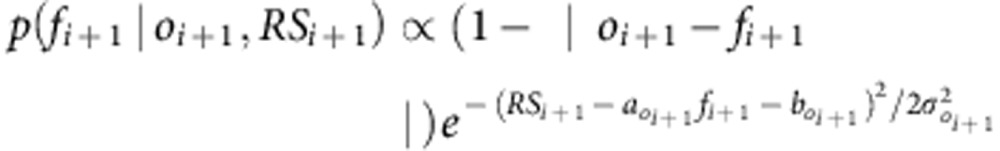


Where |*o*_*i*+1_–*f*_*i*+1_| quantified the discrepancy between estimated and actual conflict. RS_*i*+1_ is the reaction speed (that is, 1/RT_*i*+1_). RS_*i*+1_ was used because of its normality, an assumption that we confirmed with tests for normality of RS for each congruency condition in each subject using Kolmogorov–Smirnov tests (False discovery rate corrected *P*>0.05, indicating that the distributions do not significantly differ from Gaussian distribution). 
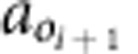
, 
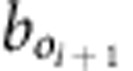
 and 
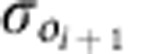
 are congruency-specific hyper-parameters that predicted RS based on a normal distribution whose mean is a function of *f*_*i*+1_ 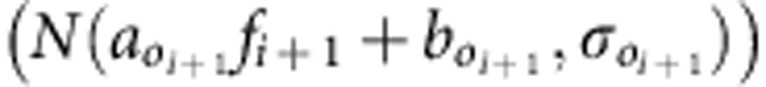. In other words, because *o*_*i*+1_ can be either 0 (congruent trial) or 1 (incongruent trial), 
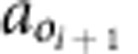
, 
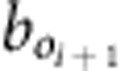
 and 
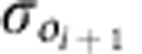
 refer to one set of hyper-parameters for each congruency type. The predicted RS was calculated using the hyper-parameters based on *o*_*i*+1_. These hyper-parameters were optimized via the expectation-maximization algorithm (Supplementary Methods). Thus 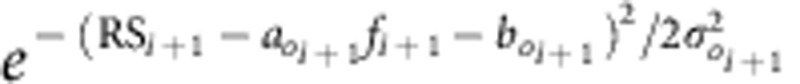 measures unsigned prediction error of RS. Finally, note that the probabilistic distribution of LRs in the flexible control model can alternatively be described as a dynamic mixing of weights of several, distinct reinforcement learners with fixed LRs (refs 49, 51). The code for this model is available on request.

### Calculating the BIC

The BIC was calculated for each subject as BIC=−2ln*L*+*k*ln(*n*), where *n* is the number of trials analysed, *k* is the number of free parameters (0, 1, 2 for the flexible control model, the reinforcement learners with one and two LRs, respectively), and *L* is the maximized value of the likelihood function. The first term (−2ln*L*) can be approximated by 
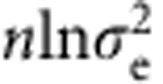
, where 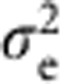 is the error variance^52^. For the model comparison analysis using observed congruency, we also ran an analysis where the likelihood for each trial was calculated as 1–|*o*_*i*_–*f*_*i*_|. For example, given a predicted conflict level of 0.8, the likelihood is 0.2 and 0.8 if the trial is congruent and incongruent, respectively. This approach yielded qualitatively equivalent model comparison results (data not shown).

### Image acquisition and preprocessing

Images were acquired parallel to the AC-PC line on a 3T GE scanner (Milwaukee, WI). Structural images were scanned using a T1-weighted SPGR axial scan sequence (146 slices, slice thickness=1 mm, TR=8.124 ms, FoV=256 mm × 256 mm, in-plane resolution=1 mm × 1 mm). Functional images were scanned using a T2*-weighted single-shot gradient EPI sequence of 39 contiguous axial slices (slice thickness=3 mm, repetition time (TR)=2 s, TE=28 ms, flip angle=90°, field of view (FoV)=192 mm × 192 mm, in-plane resolution=3 mm × 3 mm). Functional data were acquired in eight runs of 240 images each. Preprocessing was done using SPM8 ( http://www.fil.ion.ucl.ac.uk/spm/). After discarding the first five scans of each run, the remaining images were realigned to their mean image and corrected for differences in slice-time acquisition. Each subject's structural image was co-registered to the mean functional image and normalized to the Montreal Neurological Institute template. The transformation parameters of the structural image normalization were then applied to the functional images. Normalized functional images were kept in their native resolution.

### Analysing neural encoding of model variables and their interactions

To gauge the trial-wise activation in the fMRI data, a task model was built for each run. A task model consisted of regressors representing the onset of each non-excluded trial, along with five nuisance regressors representing the onsets of each type of excluded trial (error trials post-error trials, outlier trials (determined using the same criteria as in the behavioural analysis), post-outlier trials and burn-in trials) and two other nuisance regressors separately encoding onsets of left and right button-presses. This task model was then convolved with SPM 8's canonical hemodynamic response function. The convolved task model was appended by regressors representing head motion parameters and the grand mean of the run (to remove the run-specific baseline signal) to form a design matrix, against which the normalized functional images were regressed. The resulting activation maps were then concatenated across runs. As the final output of preprocessing, each grey matter voxel obtained an activation vector that chronologically represented its activation level at each trial (that is, *t*-values derived from the regressor representing that trial). The grey matter voxels were selected based on a grey matter mask, which resulted from dilating (by one voxel) voxels whose grey matter value is >0.01 in the segmented T1 template. These activation vectors were used for fMRI analyses below.

Both univariate analysis and multi-variate pattern analysis (MVPA) were carried out with a searchlight approach^53^ that scanned through small clusters (radius: 2 voxels) of grey matter voxels (Fig. 1b). Within each searchlight, the univariate analysis assessed homogeneous encoding by amplitude by fitting the variable vector to the searchlight mean activation vector. The univariate analysis was conducted via a two-fold averaging approach between the first and last four scanning runs. Each half contained two volatile runs and two stables runs (one for each proportion of incongruent trials) and was tested separately. The results were averaged across the two halves to reduce the impact of outliers. The MVPA quantified distributed encoding by the amount of signal variance in a model variable vector that could be explained by the mean-centred activation vectors from all voxels in a given searchlight through linear regression. The removal of searchlight mean signals renders the MVPA independent from the univariate analysis. Over-fitting was controlled for by a two-fold cross-validation scheme between the first and last four scanning runs. To ensure the unique attribution of fMRI signal to a given variable, all other variable and interaction vectors were used as nuisance variables in both analyses.

For each analysis, the quantification of encoding (that is, the mean *z* value of the two-fold cross validation for the multivariate analysis, or the mean *z* value across the two folds in the univariate analysis) was mapped to the centre voxel of each searchlight to form a spatial map of information content. The map was then smoothed using a Gaussian kernel of 6 mm (2 voxels) radius. One-sample *t*-tests were then conducted across individual maps to test for group-level (random) effects of homogeneous encoding and distributed encoding. To ensure that the assumptions of the *t*-tests were met, note that (1) for each searchlight, the *z* values are independently sampled from the subjects, thus fulfilling the assumption of independent sampling; and (2) the *z*-scores were derived from Fisher's *z*-transform and followed a Gaussian distribution, such that the smoothed and averaged *z*-scores are also Gaussian-distributed, fulfilling the *t*-test's assumption of a normal distribution.

Statistical results were corrected for multiple comparisons at *P*<0.05 for combined searchlight classification accuracy and cluster extent thresholds, using the AFNI ClustSim algorithm ( http://afni.nimh.nih.gov/pub/dist/doc/program_help/3dClustSim.html). Specifically, 10,000 Monte Carlo simulations were conducted, each generating a random statistical map based on the smoothness of the map resulting from the aforementioned group-level *t*-tests. For each randomly generated map, the algorithm searched for clusters using a voxel-wise *P* value threshold of <0.01. The identified clusters were then grouped to produce a null distribution of cluster size. As a result, the ClustSim algorithm determined that an uncorrected voxelwise *P* value threshold of <0.01 in combination with a searchlight cluster size 30 to 35 searchlights (depending on the specific contrast) ensured a false discovery rate of <0.05. This approach conforms to recent recommendations on statistical analysis and multiple testing correction in MVPA^54^.

## Additional information

**How to cite this article:** Jiang, J. *et al*. An insula-frontostriatal network mediates flexible cognitive control by adaptively predicting changing control demands. *Nat. Commun.* 6:8165 doi: 10.1038/ncomms9165 (2015).

## Supplementary Material

Supplementary Information Supplementary Figure 1, Supplementary Tables 1-7 and Supplementary Methods and Supplementary References
