# Supplementary material for: An insula-frontostriatal network mediates flexible cognitive control by adaptively predicting changing control demands
Source: Nat Commun. 2015 Sep 22;6:8165. doi: 10.1038/ncomms9165 (PMC4595591; doi:10.1038/ncomms9165)
Supplement: Supplementary Information — Supplementary Figure 1, Supplementary Tables 1-7 and Supplementary Methods and Supplementary References [file ncomms9165-s1.pdf]

Supplementary materials

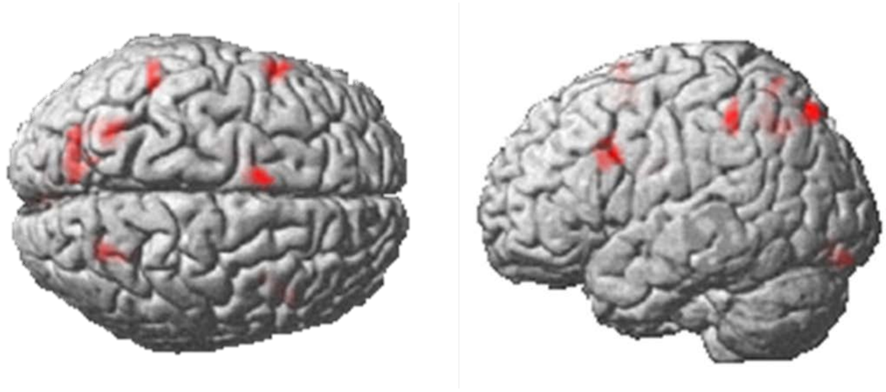

Supplementary Figure 1. Brain regions (in red, P < 0,05, whole-brain corrected) displaying significant univariate coding of observed congruency, with higher activation for incongruent than for congruent trials.

		Blocks of 80% Inc trials		Blocks of 20% Inc trials	
		Con trials	Inc trials	Con trials	Inc trials
Accuracy	Stable runs	95.4%±1.5	93.5%±1.4	93.8%±1.9	91.7%±1.8
	Volatile runs	95.4%±1.2	94.9%±1.0	95.2%±0.9	92.3%±1.1
Reaction time (in ms)	Stable runs	417±23	455±28	414±23	458±32
	Volatile runs	423±24	459±27	417±23	458±23

Supplementary Table 1. Descriptive statistics (mean ± mean standard error) of behavior, presented as a function of experimental conditions. Con = congruent; Inc = incongruent.

Flexible control model	Reinforcement learning with one fixed learning rate	Reinforcement learner with two fixed learning rates
-636.5	-633.7	-627.1
-670.0	-666.5	-660.5
130.3	131.6	137.9
-142.0	-135.3	-129.0
-533.3	-539.7	-533.5
-654.2	-647.7	-641.3
-255.2	-255.1	-248.6
-634.5	-632.8	-626.1
-441.0	-439.6	-433.1
-582.7	-577.8	-572.0
-420.0	-414.4	-408.1
-358.7	-383.9	-377.5
-564.7	-559.3	-552.9
-437.9	-432.7	-426.4
-884.9	-880.7	-874.3
-125.6	-120.5	-115.8
-337.4	-319.1	-313.2
-44.1	-41.5	-37.3
-402.3	-399.6	-393.5
-317.6	-306.8	-301.0
-147.4	-146.9	-139.8

Supplementary Table 2. Individual BICs for each of the 3 candidate models in the model comparison analysis of RT.

Flexible control model	Reinforcement learning with one fixed learning rate	Reinforcement learner with two fixed learning rates
-936.3	-897.6	-907.2
-952.4	-909.4	-913.6
-689.8	-650.9	-656.2
-929.7	-889.4	-896.5
-918.9	-891.6	-901.7
-932.7	-895.7	-908.7
-904.1	-871.3	-878.7
-963.6	-912.8	-925.8
-792.4	-769.9	-772.8
-841.1	-799.5	-803.4
-883.3	-848.2	-855.6
-706.7	-680.8	-689.4
-896.7	-866.6	-880.5
-841.3	-807.1	-817.1
-940.4	-909.1	-918.1
-724.4	-680.0	-690.1
-908.8	-880.9	-890.7
-918.0	-875.1	-887.6
-971.0	-935.8	-948.1
-901.0	-891.5	-902.2
-925.0	-919.8	-926.4

Supplementary Table 3. Individual BICs for each of the 3 candidate models in the model comparison analysis of congruency.

Location	Peak MNI	Peak beta	Peak t-value	Cluster size (# searchlights)
**Encoding of congruency (univariate)**				
L. Lingual gyrus	(0,-88,-17)	0.25	3.39	81
L. Caudate, L anterior cingulate gyrus	(-18,-4,25)	0.28	4.12	55
R. Inferior frontal gyrus	(39,11,12)	0.08	3.28	36
L. Inferior frontal gyrus	(-60,11,31)	0.18	4.61	56
R. Precuneus, R. Superior occipital gyrus	(21,-64,43)	0.24	3.75	57
L. Precuneus, L. Superior occipital gyrus	(-27,-61,43)	0.48	4.87	265
L. Inferior parietal lobule	(-54,-40,46)	0.27	3.81	37
L. Supplementary motor area	(-9,2,58)	0.45	4.75	84
**Encoding of predicted conflict level (univariate)**				
L. Inferior parietal lobule	(-48,-40,58)	0.80	4.12	118
L. Superior frontal gyrus, L. Middle frontal gyrus	(30,2,64)	1.00	3.72	133
R. Postcentral Lobule	(3,-25,67)	0.72	3.55	53
R. Paracentral Gyrus	(24,-31,73)	0.78	2.98	33
**Encoding of predicted conflict level (multivariate)**				
L. Inferior temporal gyrus	(-42,-61,-8)	0.83	5.33	65
R. Caudate	(12,5,16)	0.84	4.19	56
L. Insula, L. Postcentral gyrus	(-42,-12,22)	0.82	4.50	51
**Encoding of flexible LR/volatility (univariate)**				
R. Parahippocampal gyrus, R. Amygdala, R. Putamen	(18,-13,-23)	0.56	3.85	51
R. Insula, R. Inferior frontal gyrus	(24,11,-17)	0.47	3.97	50
L. Insula, L. Inferior frontal gyrus	(-24,-1,-5)	0.50	3.61	77
R. Precuneus	(12,-58,22)	0.68	3.62	73
L. Superior occipital gyrus	(-24,-97,22)	0.31	3.63	44

Supplementary Table 4: Summary of clusters showing significant univariate/multivariate encoding of model variables.

	p	α	ep	p×c	α×c	α×p	α×p×c
c	0.143	2.6e-4	0.0027	1.1e-6	1.7e-9	0.0034	0.0012
p	-	0.0014	0.0043	0.0017	0.0036	0.0430	0.0029
α	-	-	0.0554	0.0042	0.0071	0.0208	0.1104
ep	-	-	-	0.0502	0.0074	0.0087	0.0038
p×c	-	-	-	-	0.0014	0.0023	0.0407
α×c	-	-	-	-	-	0.1099	0.0206
α×p	-	-	-	-	-	-	0.0187

Supplementary Table 5: Group mean r^2 between model estimates of variables/interactions. c: observed congruency; p: predicted conflict level; α: flexible learning rate; ep: uncertainty of predicted conflict level. All variables/interactions were normalized within each subject to avoid bias caused by different ranges of variable/interactions.

	p	α	ep	p×c	α×c	α×p	α×p×c
c	4e-28	n.s.	n.s.	n.s.	n.s.	n.s.	n.s.
p	-	n.s.	n.s.	n.s.	n.s.	2e-8	n.s.
α	-	-	8e-11	n.s.	n.s.	0.029	3e-20
ep	-	-	-	2e-8	n.s.	n.s.	n.s.
p×c	-	-	-	-	n.s.	n.s.	4e-8
α×c	-	-	-	-	-	4e-20	0.031
α×p	-	-	-	-	-	-	0.0031

Supplementary Table 6: Group median P-values of within-subject correlation between model estimates of variables/interactions. c: observed congruency; p: predicted conflict level; α: flexible learning rate; ep: uncertainty of predicted conflict level. All variables/interactions were normalized within each subject to avoid bias caused by different ranges of variable/interactions. Note that due to the high degrees of freedom (~600 trials), highly significant correlation does not guarantee high shared variance. n.s.: not significant.

	a	b	σ
Congruent trials	-0.21±0.09	2.80±0.14	0.67±0.04
Incongruent trials	-0.06±0.08	2.59±0.11	0.74±0.05

Supplementary Table 7: Estimates of hyper-parameters (group mean ± mean standard error).

Supplementary Methods

The interaction between Predicted Conflict Level and Congruency as a measure of control prediction error

After normalization, the congruent and incongruent trials were represented as -1 and 1, respectively. The normalized predicted conflict level f_i represents the (belief of the) probability of encountering an incongruent trial. Assuming f_i is centered and re-scaled between -1 and 1, the unsigned prediction error of congruency can then be quantified using the negative of the interaction term between predicted conflict level and congruency, $-f_i \times o_i$. The unsigned prediction error of congruency, formally formulated as:

$$\begin{cases} 1 - f_i, \text{if } o_i = 1 \\ 1 + f_i, \text{if } o_i = -1 \end{cases}$$

can be further re-formulated to $1 - f_i \times o_i$. In the context of a regression analysis, the constant 1 can furthermore be discarded without affecting the results. In fact, although the normalized f_i was not re-scaled between -1 and 1 in the analyses, $-f_i \times o_i$ can still be used as prediction error multiplied by the re-scaling factor.

Estimating Hyper-parameters in the Flexible Control Model

For each subject, the hyper-parameters a, b and σ (one set for each congruency condition) were estimated using the expectation-maximization algorithm[1]. a and b were initialized by fitting the estimates of predicted conflict level, which were generated by the flexible control model using only the observed congruency, to the RS. The linear model used in fitting is the same as how the flexible control model generates RS from predicted conflict level, namely:

$$RS_{i+1} = \mathrm{a}_{o_{i+1}}\mathrm{f}_{\mathrm{i+1}} + \mathrm{b}_{o_{i+1}}$$

σ was initialized as the standard deviation of the residual of the fit. Then the following E and M steps were repeated until the estimated hyper-parameters converged:

E step: Applying a, b and σ to the flexible control model to generate the sequences of α and f using both congruency and RS.

M step: Updating a, b and σ using the sequence of f generated in the E step and the same procedure as the initialization.

Note that the hyper-parameters remained constant during the estimation of model parameters. Thus the omission of the normalization factor in the Gaussian distribution was remedied by normalization of probabilistic distributions at the end of the E step.

As expected, the baseline RS (i.e., $0.5\mathrm{a}_\mathrm{i} + \mathrm{b}_\mathrm{i}$, the predicted RS when the forthcoming trial is believed to be equally likely to be congruent or incongruent) was significantly faster in congruent than in incongruent trials (t_{20} = 3.34, P < 0.005, Supplementary Table 7).

Estimating neural LRs

We first extracted the neural coding of predicted conflict level, and then analyzed the LRs estimated from the neural data. Specifically, for each searchlight in the candidate brain regions identified in the analysis of the conflict prediction model variable vector, its GM voxels' activation vectors were applied to fit the variable vector of congruency using linear regression, and the fitted activation vector (i.e., the regression coefficient-weighted sum of activation vectors) was used as the model-free neural coding vector of predicted conflict level. This is based on the fact that the linear regression minimized the sum of square error between the observed congruency and the predicted conflict level, and hence the fitted activation vector can be considered as the searchlight's best approximation of congruency and best prediction of conflict level. In the fitted activation vector, values were hard-thresholded to the range from 0 to 1 (the probability of conflict). The ROI mean model-free neural coding vector of predicted conflict level average was then used to estimate LRs based on a standard reinforcement learning algorithm[2], which takes the following form:

$$\widetilde{f_{i+1}} - \widetilde{f_i} = \alpha(o_i - \widetilde{f_i})$$

where $\widetilde{f_i}$ is the neural coding of predicted conflict level at trial i, and α is the LR. In other words, the reinforcement learning algorithm defines a linear mapping from the prediction error $o_i - \widetilde{f_i}$ to the updating of neural coding of predicted conflict level $\widetilde{f_{i+1}} - \widetilde{f_i}$. Thus, with o_i known and $\widetilde{f_i}$ and $\widetilde{f_{i+1}}$ estimated, α can then be estimated across trials by a linear regression. To test the modulation of volatility on neural LRs, an updated linear model was applied to the neural coding of predicted conflict level:

$$\alpha = 1 + \beta v_{i+1}$$

This new linear model defines a linear correlation between the α and the volatility v_{i+1}. This model was then applied to estimating β, which was further tested against 0 using a one-sample t-test across subjects to examine if higher volatility leads to higher neural LR.

Validating model components

At the core of the flexible control model lies the assumption that human performance is driven by learned predictions of control demand. To ensure that our model's ability to perform this type of learning actually facilitates a better explanation of human behavior, we performed an additional model comparison between the flexible control model and a reduced model with no conflict learning ability (i.e., here the learning rate was set to 0 for all trials), using the BIC. Note that because the absence of learning renders the predicted conflict level constant across all trials, fitting observed congruency and RT to this reduced model is equivalent to fitting these observations to two Gaussian distributions (one for each congruency condition). In line with the basic premise that human subjects learn to predict control demand during task performance, we found that the flexible control model greatly outperformed the reduced model (exceedance probability = 0.95).

To ensure that this propagation process indeed boosts the flexible control model's ability to predict control-demand and account for behavioral data, we repeated the model comparison analysis between the full flexible control model and a reduced flexible control model without the propagation process, using the BIC. While both models performed similarly in terms of explaining RTs (exceedance probability = 0.42 for the flexible control model), the flexible control model was far superior in accounting for the observed congruency (exceedance probability = 0.96). Thus, the inclusion of the propagation process significantly improves the flexible control model's performance.

To validate the inclusion of the RT as a tool for inferring internal states in the flexible control model, we conducted a model comparison analysis between the full flexible control model and a reduced flexible control model that did not use RT for inference, using the BIC. We found that the full model significantly outperformed the reduced model in explaining observed congruency (exceedance probability = 0.76), possibly due to the fact that RTs show a congruency effect (see above) and hence provide additional information to help predict forthcoming conflict. An additional model comparison between the same models to account for RT (for the reduced model, the predicted RTs were produced by running the initialization step of the EM algorithm, see Supplementary Methods) revealed that the full flexible control model was also better at explaining the variance in RTs (exceedance probability = 0.96). Thus we conclude that the inclusion of RT significantly improves the performance of the flexible control model.

Computational implementation of the flexible control model

In the computational implementation of the flexible control model, the joint distribution of k, α, f was represented in a 3D grid, with each dimension representing one variable and each cell representing the probability of this distribution at the point (k, α, f). k ranged from 0.04 to 1 with an interval of 0.04; f ranged from 0 to 1 with an interval of 0.02. Regarding the representation of α, it has been shown that the beta distribution in the propagation procedure can be interpreted as a likelihood function of conflict level (after the propagation) based on the belief of conflict level f (before the propagation) and the number of trials used for learning (i.e., $1/\alpha$)[3]. This reflects the assumption that in a more stable environment, the learning rate is lower and the learning of conflict level can be based on more trials in the recent trial history. Additionally, a lower α also leads to a beta distribution with smaller variance, hence reflecting the assumption that in a more stable environment the belief of conflict level is more precise. Thus we set the possible range of $1/\alpha$ from 1 to 160 (i.e., the number of non-burn-in trials up to 2 runs), and equally divided them into 80 points. This representation of α is denser when α is lower. This is also in line with previous finding that the learning rate tends to be low (around 0.05) in order to best learn the proportion congruency[4]. This joint distribution was initialized as a uniform distribution. Similarly, other distributions were represented in grids. All computations in this model were conducted on these grids. All computation in this model can be (approximately) implemented between points in the grids using weighted sums, a biologically feasible operation that has been used in many computational models of neuronal activity[5]. Additionally, the biological feasibility of maintaining a joint probabilistic distribution is supported by recent work on neural encoding of probabilistic distributions[6] and multidimensional information[7].

Supplementary references

1. Dempster, A.P., Schatzoff, M. & Wermuth, N. Simulation Study of Alternatives to Ordinary Least-Squares. *Journal of the American Statistical Association* **72**, 77-93 (1977).
2. Sutton, R.S. Learning to predict by the method of temporal dierences. *Machine Learning* **3**, 9-44 (1988).
3. Jiang, J., Heller, K. & Egner, T. Bayesian modeling of flexible cognitive control. *Neuroscience and biobehavioral reviews* (2014).
4. Botvinick, M.M., Braver, T.S., Barch, D.M., Carter, C.S. & Cohen, J.D. Conflict monitoring and cognitive control. *Psychological review* **108**, 624-652 (2001).
5. Dayan, P. & Abbott, L.F. *Theoretical Neuroscience* (The MIT Press, Cambridge, Massachusetts

London, England, 2001).
6. Pouget, A., Beck, J.M., Ma, W.J. & Latham, P.E. Probabilistic brains: knowns and unknowns. *Nature neuroscience* **16**, 1170-1178 (2013).
7. Eurich, C.W. & Wilke, S.D. Multidimensional encoding strategy of spiking neurons. *Neural computation* **12**, 1519-1529 (2000).
